# HER2 genomic amplification in circulating tumor DNA and estrogen receptor positivity predict primary resistance to trastuzumab emtansine (T-DM1) in patients with HER2-positive metastatic breast cancer

**DOI:** 10.1007/s12282-018-0861-9

**Published:** 2018-04-26

**Authors:** Hitomi Sakai, Junji Tsurutani, Tsutomu Iwasa, Yoshifumi Komoike, Kazuko Sakai, Kazuto Nishio, Kazuhiko Nakagawa

**Affiliations:** 1 Department of Medical Oncology, Kindai University Faculty of Medicine, 377-2 Ohno-higashi, Osaka-Sayama, Osaka Japan; 2 Department of Surgery, Kindai University Faculty of Medicine, 377-2 Ohno-higashi, Osaka-Sayama, Osaka Japan; 3 Department of Genome Biology, Kindai University Faculty of Medicine, 377-2 Ohno-higashi, Osaka-Sayama, Osaka Japan

**Keywords:** HER2, Trastuzumab emtansine (T-DM1), Primary resistance, Circulating tumor DNA (ctDNA)

## Abstract

**Background:**

Trastuzumab emtansine (T-DM1) is approved for the treatment of patients with human epidermal growth factor receptor 2 (HER2)-positive advanced breast cancer (ABC), and has high efficacy. However, some patients exhibit primary resistance to T-DM1, and thus methods that can predict resistance in clinical practice are needed. Genomic analysis of circulating tumor DNA (ctDNA) in plasma is a non-invasive and reproducible method. This study aimed to predict primary resistance to T-DM1 by combining genomic analysis of ctDNA and other clinicopathological features of patients with HER2-positive ABC.

**Methods:**

The study population comprised 34 patients with HER2-positive ABC who had been treated with T-DM1. Correlations between clinicopathological characteristics of patients and primary resistance to T-DM1 were examined, and *HER2* gene copy number and *PIK3CA* gene mutations were analyzed using plasma ctDNA samples obtained from 16 patients before T-DM1 administration.

**Results:**

Among the 34 patients, nine (26.5%) had progressive disease at the first efficacy analysis; these patients were considered to have primary resistance to T-DM1. No significant difference was found in the rate of primary resistance to T-DM1 between groups. Among 16 patients whose ctDNA was analyzed, four showed primary resistance to T-DM1. These four patients showed negative HER2 gene amplification in ctDNA and were ER-positive and/or PR-positive by immunohistochemistry.

**Conclusions:**

HER2 gene amplification in ctDNA and ER and PR status may predict primary resistance to T-DM1. A liquid biopsy before the initiation of T-DM1 treatment could be a non-invasive way to predict whether a patient would exhibit primary resistance to T-DM1.

## Introduction

T-DM1 is an antibody–drug conjugate that combines the human epidermal growth factor receptor type 2 (HER2)-targeted antitumor properties of trastuzumab with the maytansinoid, DM1, a potent microtubule-disrupting agent, joined by a stable linker [1]. T-DM1 significantly improved progression-free survival (PFS) and overall survival (OS) compared to the combination of capecitabine and lapatinib in the phase III EMILIA study [2], and has become a standard treatment for patients with HER2-positive ABC previously treated with trastuzumab and taxanes in accordance with ASCO guidelines [3]. In the phase III MARIANNE trial, patients with previously untreated HER2-positive locally advanced or metastatic breast cancer were randomized to trastuzumab plus a taxane (docetaxel or paclitaxel), T-DM1 plus placebo, or T-DM1 plus pertuzumab. Both T-DM1-containing regimens showed non-inferiority, but not superiority, in terms of PFS over trastuzumab plus a taxane. The objective response rate was 67.9% with trastuzumab plus a taxane, 59.7% with T-DM1 plus placebo, and 64.2% with T-DM1 plus pertuzumab [4]. These results implied that a significant proportion of patients experience disease progression without any signs of tumor regression or stabilization of disease despite early treatment with T-DM1, and the primary resistance rate to T-DM1 is probably as high as 30%. In contrast, patients whose tumors responded to T-DM1 tended to have durable responses with favorable toxicity profiles and QOL compared to the comparator [4]. Thus, if patients with tumors that are primarily resistant to T-DM1 are excluded, the drug could have a greater therapeutic index.

Several mechanisms of resistance to HER2-directed therapies have been described in preclinical studies, including the accumulation of 95HER2, truncated ERBB2 receptors which lack an extracellular trastuzumab-binding domain [5], loss of PTEN which activates the phosphoinositide 3-kinase (PI3K) pathway [6], mutations in phosphoinositide-3-kinase, catalytic, alpha polypeptide (*PIK3CA*) [7, 8], heregulin-dependent-HER2/HER3 signaling [9, 10], and signaling through insulin-like growth factor 1 receptor [11]. However, low expression of HER2 mRNA or HER2 protein is the only clinically definite biomarker related to low efficacy of T-DM1 [12, 13]. Methods of predicting drug resistance that are feasible in clinical settings are highly desired.

Cell-free DNA (cfDNA) refers to fragmented DNA from cells that circulate in blood [14–16]. cfDNA in cancer patients include tumor-derived DNA (circulating tumor DNA: ctDNA), which can harbor tumor-specific genetic changes. Genomic analysis of ctDNA in plasma is non-invasive and reproducible, and can be used to elucidate mechanisms underlying drug resistance.

This study aimed to predict primary resistance to T-DM1 by combining genomic analysis of ctDNA and other clinicopathological features of patients with HER2-positive ABC.

## Patients and methods

### Patients and data collection

Electronic medical records at Kindai University Hospital were used to identify breast cancer patients who received T-DM1 from May 1, 2015 to April 30, 2017 at the Department of Surgery or Department of Medical Oncology. There were 34 eligible patients and no patient was excluded. Therefore, 34 patients were included in the study. The following data were collected: patient demographics, including date of birth and sex; diagnoses; and tumor characteristics, including stage (TNM classification, 7th edition), pathological diagnosis, estrogen receptor (ER) status, progesterone receptor (PR) status, and HER2 status. Hormonal receptor-positive samples were defined as those in which ≥ 10% of cells were positive by immunohistological staining. HER2 positivity was assessed by immunohistochemistry (IHC). In cases with IHC2+, the fluorescent in situ hybridization (FISH) test was performed. Treatment history was also collected, including information on neoadjuvant or adjuvant treatment regimens, chemotherapy and hormonal therapy before T-DM1 for metastatic disease and tumor response, and number and type of chemotherapy for metastatic breast cancer. Tumor response to treatment was determined by the treating physician using Response Evaluation Criteria in Solid Tumors (RECIST). In cases of recurrent metastatic disease, the first chemotherapy administered was defined as first-line chemotherapy, whereas in cases of primary stage IV disease, overall first chemotherapy was defined as first-line chemotherapy. Maintenance anti-HER2 therapy was not counted as a treatment. For example, paclitaxel plus trastuzumab followed by trastuzumab was counted as one regimen.

The date of T-DM1 treatments and tumor response to T-DM1 using computed tomography (CT) and RECIST v1.1 [17] were recorded. A patient was considered to have primary resistance to T-DM1 when progressive disease (PD) was recorded in the first evaluation of T-DM1 response.

This study was approved by the ethics committee of Kindai University Faculty of Medicine. Written informed consent is mandatory for collecting ctDNA at our institution. However, patients were given the opportunity to opt-out of ctDNA collection and provide only information via a website.

### Circulating tumor DNA analysis

*HER2* gene copy number and *PIK3CA* gene mutations were analyzed using ctDNA from 16 patients who provided written informed consent before T-DM1 administration. Seven milliliters of venous blood were collected before the patients were administered T-DM1. EDTA-treated blood samples were centrifuged and plasma supernatant was stored at − 80 °C until analysis. DNA was purified from plasma with the cobas® ctDNA Sample Preparation Kit (Roche Diagnostics, Pleasanton, CA). The copy number of *HER2* and *PIK3CA* mutations was determined using the QX100 Droplet Digital PCR System in accordance with the manufacturer’s instructions (Bio-Rad, Hercules, CA, USA). The relative *HER2* copy number ratio was calculated relative to the control Elongation factor Tu GTP-binding domain 2 (*EFTUD2*), as previously described [18].

Primer sequences used in the *HER2* copy number analysis were as follows: HER2 forward, 5′-ACAACCAAGTGAGGCAGGTC-3′; HER2 reverse, 5′-GTATTGTTCAGCGGGTCTCC-3′; HER2 probe, 5′-/56-FAM/AGGCACCCA/ZEN/GCTCTTTGAGGACAAC/3IABkFQ/-3′; EFTUD2 forward, 5′-GGTCTTGCCAGACACCAAAG-3′; EFTUD2 reverse, 5′-TGAGAGGACACACGCAAAAC-3′; EFTUD2 probe, 5′-/5HEX/TCCAGGTAG/ZEN/GACATCCTTTGGCTTT/3IABkFQ/-3′ [18].

PCR was performed using the following cycling conditions: 95 °C for 10 min, 40 cycles of 94 °C for 30 s and 60 °C for 60 s, followed by enzyme deactivation at 98 °C for 10 min. For the *PIK3CA* mutation assay, primers and probes for detecting *PIK3CA* mutations E542K, E545K, and H1047R were purchased from Bio-Rad. PCR was performed using the following cycling conditions: 95 °C for 10 min, 40 cycles of 94 °C for 30 s and 55 °C for 60 s, followed by enzyme deactivation at 98 °C for 10 min. After thermal cycling, plates were transferred to a Droplet reader. Digital PCR data were analyzed using the QuantaSoft analytical software package (Bio-Rad). The copy number of each gene was estimated from the Poisson distribution. Positive HER2 amplification with digital PCR was defined as a HER2 ratio (HER2/EFTUD2 copy number ratio) of 1.25, as described in a previous study [18].

### Minimum detection limit of the assay and determination of cut-off values

The digital PCR method showed minimum detection limits of 0.03% for *PIK3CA* E542K, 0.03% for E545K, and 0.03% for H1047R using plasmid DNA (data not shown). For plasma samples, cut-off values were determined using samples from healthy volunteers. The cut-off value for plasma ctDNA samples was set at 3 copies for all *PIK3CA* mutations, because the background noise in plasma ctDNA from 10 healthy volunteers was exactly zero. For *HER2* copy number, the cut-off value for plasma ctDNA samples was set at 1.25, as described in a previous study [18].

### Statistical analysis

Descriptive statistics were used to examine demographics of patients and tumor characteristics. Fisher’s exact test was used to compare the rate of primary resistance to T-DM1 in two independent groups. Time to progression (TTP), which was defined as the number of days from T-DM1 initiation to objective disease progression, was estimated by the Kaplan–Meier method. An unstratified log-rank test was used to compare TTP between groups.

All tests were two tailed, with *p* < 0.05 considered statistically significant. All survey data were coded and analyzed with the use of standard EZR (Saitama Medical Center, Jichi Medical University), a graphical user interface for R (The R Foundation for Statistical Computing, version 2.13.0). More precisely, it is a modified version of R commander designed to add statistical functions frequently used in biostatistics [19].

## Results

### Patients’ characteristics and response to T-DM1

A total of 34 patients with HER2-positive metastatic breast cancer who had been treated with T-DM1 were included in this study. Plasma samples were obtained from 16 patients who provided written informed consent for ctDNA analysis. Demographic and disease characteristics of the study population are summarized in Table 1. Only 26.5% of patients had primary stage IV breast cancer. HER2 status of the primary site was IHC 3+ in 30 patients (88.2%) and IHC 2+ and FISH positive in four patients (11.8%). Of these 34 patients, 52.9% had ER-positive and/or PR-positive breast cancer. Fifty percent of patients were administered T-DM1 as first- or second-line chemotherapy for metastatic disease. All but one patient had received T-DM1 in the neoadjuvant, adjuvant, or metastatic setting. Table 1 also summarizes prior therapies (before T-DM1) for metastatic disease.

**Table 1:** Demographic and disease characteristics of the study population (*N* = 34)

Characteristics	No. (%)
Age, years	
Median (range)	62 (38–84)
Stage IV or recurrent	
Stage IV	9 (26.5)
Recurrent	25 (73.5)
Pathology	
Invasive ductal carcinoma	32 (94.1)
Medullary carcinoma	1 (2.9)
Poorly differentiated adenocarcinoma	1 (2.9)
*HER2* status of primary site	
IHC 3+	30 (88.2)
IHC 2+ and FISH+	4 (11.8)
Hormone receptor status	
ER and/or PR positive	18 (52.9)
ER and PR negative	16 (47.1)
Neoadjuvant or adjuvant chemotherapy	
Anthracycline followed by taxane + trastuzumab	8 (23.5)
Taxane + trastuzumab	4 (11.8)
Trastuzumab without chemotherapy	2 (5.9)
Anthracycline followed by trastuzumab	1 (2.9)
Anthracycline followed by taxane	1 (2.9)
Taxane	1 (2.9)
UFT + trastuzumab	1 (2.9)
CMF	1 (2.9)
Doxifluridine	1 (2.9)
Neoadjuvant or adjuvant endocrine therapy	
Yes	10 (29.4)
Prior lines of chemotherapy for metastatic disease before T-DM1	
0	2 (5.9)
1	15 (44.1)
2	4 (11.8)
3	6 (17.6)
4	3 (8.8)
≥ 5	4 (11.8)
Type of prior therapy for metastatic disease before T-DM1	
Trastuzumab	29 (85.3)
Taxane	20 (58.8)
Pertuzumab	15 (44.1)
Capecitabine	15 (44.1)
Hormonal therapy	12 (35.3)
Lapatinib	11 (32.4)
Vinorelbine	7 (20.6)
S-1	4 (11.7)
Eribulin	3 (8.8)
Cyclophosphamide	2 (5.8)
Others	4 (11.7)

*IHC* immunohistochemistry; *FISH* fluorescent in situ hybridization; *CMF* cyclophosphamide, methotrexate, and fluorouracil

All 34 patients were available for efficacy analysis. Best response to T-DM1 is shown in Table 2. The response rate was 26.5%. Nine patients (26.5%) had progressive disease at the first efficacy analysis, and were considered to have primary resistance to T-DM1.

**Table 2:** Best response to T-DM1

Response	No. of patients (%)
CR + PR	9 (26.5)
CR	2 (5.9)
PR	7 (20.6)
SD	13 (38.2)
PD	9 (26.5)
Unknown	3 (8.8)
Total	34

*CR* complete response, *PR* partial response, *SD* stable disease, *PD* progressive disease

### HER2 copy number gain and PIK3CA mutations in plasma samples

Results of *HER2* copy number and *PIK3CA* mutation analyses of plasma samples are summarized in Table 3. For both the *HER2* copy number assay and *PIK3CA* mutation assay, all plasma ctDNA samples could be evaluated, since the copy number of the reference gene (*EFTUD2*) was more than 100. *HER2* copy number gains were observed in 5 of 16 patients (31%). The *PIK3CA* E545K mutation was detected in 2 of 16 patients (13%), while the *PIK3CA* E542K mutation and H1047R mutation were not detected.

**Table 3:** *HER2* copy number gain and *PIK3CA* mutations in plasma samples

No.	Relative quantitation of *HER2* gene	*PIK3CA* E542K-MUT	*PIK3CA* E542K-WT	*PIK3CA* E545K-MUT	*PIK3CA* E545K-WT	*PIK3CA* H1047R-MUT	*PIK3CA* H1047R-WT
		(copies)					
1	0.91	0	686	0	726	0	674
2	1.1	1.6	344	0	390	1.6	398
3	0.95	0	396	0	480	0	402
4	0.93	0	202	0	222	0	186
5	1.02	0	552	0	592	0	632
6	1.11	0	1264	18^a^	1174	0	1210
7	1.11	0	814	0	864	2.4	818
8	1.06	0	612	0	642	0	508
9	2.14^a^	0	1066	0	1000	0	990
10	4.23^a^	0	572	0	628	0	556
11	0.93	1.8	4000	4.6^a^	3860	0	4140
12	1.45^a^	0	480	0	522	0	482
13	7.56^a^	0	1680	1.6	1620	0	1480
14	1.23	0	144	0	220	0	248
15	1.82^a^	0	502	0	552	0	570
16	1.14	0	512	0	540	0	544

*HER2* human epidermal growth factor receptor 2; *PIK3CA* phosphatidylinositol-4,5-bisphosphate 3-kinase, catalytic subunit alpha; *WT* wild type; *MUT* mutant

^a^Greater than cut-off value

### Predictors of primary resistance to T-DM1

The rate of primary resistance to T-DM1 was compared between the following groups: recurrent vs. primary stage IV, ER-positive and/or PR-positive vs. ER-negative and PR-negative, T-DM1 as second-line therapy or earlier vs. T-DM1 as later than second-line therapy, prior treatment with anthracycline vs. no prior treatment with anthracycline, prior treatment with pertuzumab vs. no prior treatment with pertuzumab, prior treatment with lapatinib vs. no prior treatment with lapatinib, HER2 gene amplification positive vs. negative, and *PIK3CA* mutation positive vs. negative (Table 4). Among these, those with ER-positive and/or PR-positive disease were more likely to have primary resistance to T-DM1 than those with ER-negative and PR-negative disease, but the difference between the two subsets was not significant (35.3% vs. 17.6%, *p* = 0.438). Diseases treated with T-DM1 as second-line therapy exhibited primary resistance to T-DM1 less often than those treated with T-DM1 as third- or later-line therapy, but the difference was not significant (17.6% vs. 35.3%, *p* = 0.438). Diseases with HER2 gene amplification were less likely to have primary resistance to T-DM1 compared to those without HER2 gene amplification, but this difference also was not significant (0% vs. 36.4%, *p* = 0.245). Both patients with *PIK3CA* mutations showed primary resistance to T-DM1, while only two of 14 patients without *PIK3CA* mutations showed primary resistance to T-DM1 (100% vs. 14.3%, *p* = 0.05).

**Table 4:** Clinicopathological features and primary resistance to T-DM1

Characteristics	No. of patients (%)	*p* value
All patients	34	
Primary resistance to T-DM1	9 (26.5)	
De novo stage IV or recurrent		1
De novo stage IV	9	
Primary resistance to T-DM1	2 (22.2)	
Recurrent	25	
Primary resistance to T-DM1	7 (28.0)	
Hormone receptor status		0.438
ER and/or PR positive	17	
Primary resistance to T-DM1	6 (35.3)	
ER and PR negative	17	
Primary resistance to T-DM1	3 (17.6)	
T-DM1 as second-line therapy or earlier	17	0.438
Primary resistance to T-DM1	3 (17.6)	
T-DM1 as later than second-line therapy	17	
Primary resistance to T-DM1	6 (35.3)	
Prior anthracycline		1
Yes	11	
Primary resistance to T-DM1	3 (27.3)	
No	23	
Primary resistance to T-DM1	6 (26.1)	
Prior pertuzumab		0.462
Yes	15	
Primary resistance to T-DM1	5 (33.3)	
No	19	
Primary resistance to T-DM1	4 (21.1)	
Prior lapatinib		1
Yes	11	
Primary resistance to T-DM1	3 (27.3)	
No	23	
Primary resistance to T-DM1	6 (26.1)	
*HER2* gene amplification in ctDNA		0.245
Positive	5	
Primary resistance to T-DM1	0 (0)	
Negative	11	
Primary resistance to T-DM1	4 (36.4)	
*PIK3CA* mutation in ctDNA		0.05
Mutation positive	2	
Primary resistance to T-DM1	2 (100)	
Mutation negative	14	
Primary resistance to T-DM1	2 (14.3)	

**Fig. 1:**
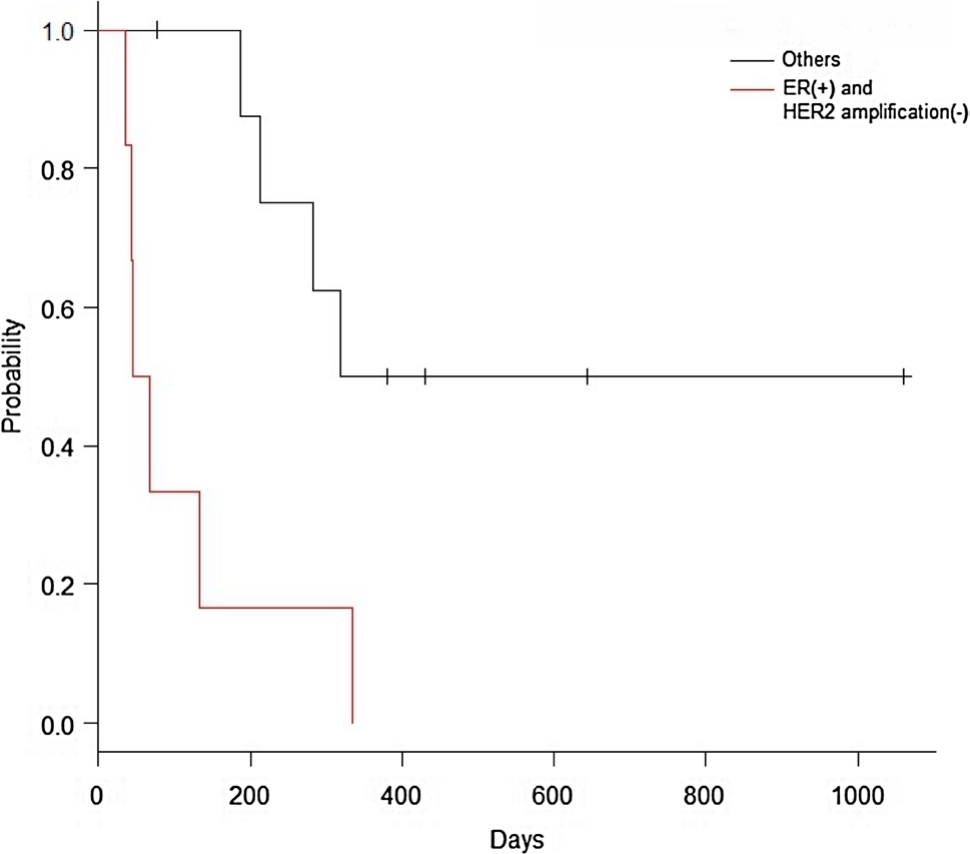
Kaplan–Meier curve for time to progression (TTP). Sixteen patients whose ctDNA was analyzed were subjected to this analysis. Red line indicates patients with ER-positive and/or PR-positive breast cancer and negative HER2 amplification (*n* = 6), with a median TTP of 56.6 days (95% CI 36-NA). Black line indicates others (*n* = 10), with a median TTP of 318.0 days (95% CI 187-NA) (*p* = 0.0037). Whiskers indicate censored observations

Among 16 patients whose ctDNA was analyzed, four showed primary resistance to T-DM1. These four patients showed negative HER2 gene amplification in ctDNA and were ER positive and/or PR positive (Table 5). Among the 16 patients, median TTP was 56.6 days (95%CI: 36-NA) for those with negative HER2 gene amplification in ctDNA and ER-positive and/or PR-positive tumors, and 318.0 days (95%CI: 187-NA) in patients with neither of these findings (*p* = 0.0037) (Fig. 1).

**Table 5:** Rates of primary resistance to T-DM1 per *HER2* gene amplification in ctDNA and hormone receptor status

		*HER2* gene amplification in ctDNA	
		Positive (*N*/total)	Negative (*N*/total)
Hormone receptor status	ER and PR negative (*N*/total)	0/4	0/5
	ER and/or PR positive (*N*/total)	0/1	4/6

*N* number of patients with primary resistance to T-DM1

## Discussion

In this study, we investigated predictive markers for primary resistance to T-DM1. Negative HER2 gene amplification in ctDNA taken just before the initiation of T-DM1 treatment, ER positivity, and/or PR positivity are candidate negative predictive biomarkers. Our results suggest the potential importance of crosstalk between ER and HER2 pathways in the resistance to T-DM1 treatment.

In exploratory analysis, although not significant, breast cancer with ER and/or PR positivity, T-DM1 administered as third- or later-line therapy, and HER2 gene amplification negativity verified by ctDNA analysis tended to exhibit primary resistance to T-DM1. The lack of significance was likely due to a lack of statistical power. Further studies should be conducted to determine whether these factors are predictive factors using a larger population.

Among patients whose ctDNA was analyzed, all patients with breast cancer who had primary resistance to T-DM1 were ER positive and/or PR positive by IHC, and HER2 amplification-negative by analysis of ctDNA obtained just before initiation of T-DM1 treatment. TTP was significantly shorter in these patients compared to their counterparts. These data support previous studies that suggested the importance of crosstalk between ER and HER2 pathways in the resistance to anti-HER2 treatment. It is well known that patients with higher *HER2* amplification have lower ER levels [20]. Moreover, crosstalk between ER and HER2 pathways is implicated in the resistance to hormonal therapy [21–23]. Preclinical and clinical data also suggest that crosstalk between ER and HER2 pathways is involved in the resistance to anti-HER2 treatment. For instance, one study reported that ER expression was significantly increased in xenograft tumors with acquired resistance to anti-HER2 therapy compared with untreated tumors in preclinical models [24]. In the phase III HERA trial, adjuvant trastuzumab followed by chemotherapy showed less benefit for HER2-positive breast cancer patients with ER-positive tumors and who had a low FISH ratio (≥ 2 to < 5) [25]. NeoALTTO, a phase III trial of neoadjuvant lapatinib with trastuzumab, showed a higher pathological complete response (pCR) rate in patients with ER-negative tumors than those with ER-positive tumors [26]. In CALGB40601, a phase III trial of neoadjuvant paclitaxel and trastuzumab with or without lapatinib, patients with ER-positive tumors showed lower rates of pCR compared to patients with ER-negative/HER2-positive tumors [27].

HER2 inhibition may enhance ER expression on tumor cells and result in T-DM1 resistance. If this hypothesis is true, then combination therapy with T-DM1 and hormonal agents may be effective in overcoming T-DM1 resistance in HER2-positive and ER-positive breast cancer. In contrast to our findings, however, in the subgroup analysis of TH3RESA, a phase III trial of T-DM1 in patients with previously treated HER2-positive metastatic breast cancer, T-DM1 was effective for both ER-positive and ER-negative breast cancer [28]. Since hormone receptors and *HER2* status can change during the course of treatment [29], hormone receptor status should also be evaluated just before treatment initiation.

In the present study, the frequency of *PIK3CA* mutations in ctDNA was too low for comparison between the *PIK3CA* mutation and wild-type groups. There has been controversy regarding the utility of *PIK3CA* mutations as a biomarker for HER2-positive breast cancer. *PIK3CA* mutations have been reported to be a prognostic factor for worse outcome in patients treated with pertuzumab, trastuzumab, and docetaxel, or capecitabine and lapatinib; however, this was not the case in T-DM1-treated patients [12, 30].

HER2 amplification was negative in 11 of 16 patients, and the concordance rate between HER2 positivity (IHC3+ or IHC2+ and FISH positive) in archival tissue and HER2 amplification in ctDNA was only 31%. The discordance in mutation profiles between ctDNA and tissue samples is generally small but evident in the setting of various cancers [31–33]. This is possibly attributed to the following three factors: time between tumor tissue sampling and blood, tumor heterogeneity, and biological factors that affect ctDNA shedding. Anti-HER2 therapy performed before liquid biopsy might contribute to changes in HER2 status. Changes in HER2 status in breast cancer after trastuzumab-based systemic therapy have been observed in several studies. One study showed that, while 17 of 23 patients with HER2 3+ (IHC) breast cancer retained HER2 3 + status, 2 and 4 patients showed HER2 2+ (IHC) and 1 + or 0 (IHC), respectively, at the primary site after neoadjuvant trastuzumab-based therapy [34]. Another study reported that 10 of 34 (43%) breast cancer patients with positive HER2 amplification by FISH at baseline became FISH negative after trastuzumab-based systemic therapy [35]. Furthermore, in a prospective study, 37% of patients with HER2-positive metastatic breast cancer (3 + by IHC or 2 + by IHC and chromogenic in situ hybridization (CISH) positive at the primary or metastatic site) lost their HER2 overexpression and were scored 0 or + 1 by IHC or negative on the CISH test after trastuzumab-based therapy; HER2 status was unchanged in the remaining cases [36]. Liquid biopsy may thus allow for non-invasive, real-time monitoring of changes in tumor biology.

This study has some limitations. First, the study was a retrospective exploratory analysis of a small number of patients at one institution. A sample size that is too small can reduce statistical power, and for this reason, we cannot draw definitive conclusion. Second, the distribution of demographic and disease characteristics differs from that of the current general population of breast cancer patients in Japan. For example, about half of our patients were heavily pre-treated before being approved for T-DM1. Moreover, five patients (14.7%) did not receive a trastuzumab-containing treatment prior to T-DM1 in the metastatic setting (three patients were administered lapatinib as anti-HER2 treatment and two received T-DM1 as first-line therapy).

In conclusion, our findings suggest that HER2 gene amplification in ctDNA and ER and PR status may predict primary resistance to T-DM1. A liquid biopsy just before the initiation of T-DM1 treatment could be a non-invasive way to predict whether a patient would exhibit primary resistance to T-DM1. Future prospective studies with a larger study population to evaluate these potential biomarkers are warranted.
